# A systematic comparison of software dedicated to meta-analysis of causal studies

**DOI:** 10.1186/1471-2288-7-40

**Published:** 2007-09-10

**Authors:** Leon Bax, Ly-Mee Yu, Noriaki Ikeda, Karel GM Moons

**Affiliations:** 1 Julius Center for Health Sciences and Primary Care, UMC Utrecht, the Netherlands; 2 Department of Medical Informatics, Kitasato University, Sagamihara, Japan; 3 Centre for Statistics in Medicine, Oxford, UK

## Abstract

**Background:**

Our objective was to systematically assess the differences in features, results, and usability of currently available meta-analysis programs.

**Methods:**

Systematic review of software. We did an extensive search on the internet (Google, Yahoo, Altavista, and MSN) for specialized meta-analysis software. We included six programs in our review: Comprehensive Meta-analysis (CMA), MetAnalysis, MetaWin, MIX, RevMan, and WEasyMA. Two investigators compared the features of the software and their results. Thirty independent researchers evaluated the programs on their usability while analyzing one data set.

**Results:**

The programs differed substantially in features, ease-of-use, and price. Although most results from the programs were identical, we did find some minor numerical inconsistencies. CMA and MIX scored highest on usability and these programs also have the most complete set of analytical features.

**Conclusion:**

In consideration of differences in numerical results, we believe the user community would benefit from openly available and systematically updated information about the procedures and results of each program's validation. The most suitable program for a meta-analysis will depend on the user's needs and preferences and this report provides an overview that should be helpful in making a substantiated choice.

## Background

Meta-analysis has been characterized in various ways, from "making order of scientific chaos"[1] to "mega-silliness"[2], and has been subject of many debates. However, time has taught – both opponents and proponents – that things are not black and white; meta-analysis, executed with care, has become an important and influential cornerstone of scientific medicine. As the quantitative part of a systematic review, the merit of meta-analysis over qualitative approaches lies in the formal and reproducible investigation of heterogeneity, small study effects, and other data trends. Although meta-analysis is applied in many types of research, the bulk of published meta-analyses are in the domain of therapeutic and – albeit to a lesser extent – observational etiologic studies. This paper focuses on this area of causal medical research and in particular the software that is being used in the corresponding meta-analyses.

Computer software has become indispensable in meta-analysis and in the last decennia many programs have been developed. To aid potential users in choosing the software that fits their needs, there are a number of reviews and comparisons available [3-7]. The most recent one, however, dates back to 5 years ago and in the meantime the spectrum of available software has changed substantially. Also, most of the existing reviews have focused on numerical features, such as which analytical models were available or what graphs could be produced. We believe that information on the validity or comparability of results and ease-of-use are equally important factors in the total applicability of the software. Therefore, the purpose of our study was to systematically compare features, results, and usability of the currently available meta-analysis software.

## Methods

### Software search and selection

We decided, a priori, to focus on software that was solely dedicated to meta-analysis of randomized therapeutic or observational causal studies. General statistics packages were excluded. Furthermore, the software had to be actively maintained and supported, which was judged by either the time of the last software update (less than 5 years), bug report (less than 5 years), or website update (less than 3 years). We also decided to select only software with a graphical interface and mouse-click compatibility, which essentially excluded the DOS programs.

Searches for software and publications related to their development and usage were done by two authors (LB, LMY) with combinations of the following keywords in Internet search engines of Google, Yahoo, AltaVista, and MSN: "meta-analysis", "meta-analyses", "systematic review", "software", "program", "package", "macro", "add-in", and "add-on". The first search was done mid 2005 and the last search in June 2006. The software was purchased or downloaded if it appeared to fulfill the inclusion criteria.

### Assessment of numerical and graphical features

The assessment of the numerical and graphical features in the included meta-analysis programs was handled independently by two investigators (LB, LMY) and reviewed by all authors until there was consensus on all items. The programs were installed and tested on Windows XP and Windows 2000 systems in English and Japanese. Details of the documented features are provided in the tables of the results section.

### Validity and comparability of meta-analysis results

We searched the internet and literature databases of medical and social sciences (PubMed, EmBase, Eric, and PsychInfo) for articles that reported validations of meta-analysis software. We also checked the website of each included program and made inquiries with its authors about their validation procedures.

In addition to the search for validation reports, two reviewers (LB, LMY) actively investigated the comparability of the numerical results with data sets from three previously published [8-10] meta-analyses (Table 1). These data sets have been used as examples in methodological meta-analysis publications [11-13] and are representative of those commonly encountered in therapeutic or etiologic meta-analyses. The first data set [8] contains per-group data from 16 randomized controlled trial articles with a dichotomous outcome, i.e. group sizes and event rates. One of the 16 included studies has no events in one of the treatment arms and the data set itself is subject to substantial small study effects. The second data set [9] contains per-group data typically found in meta-analyses of controlled trials with a continuous outcome (group sizes, means, standard deviations). It contains data from 11 studies with heterogeneous results. The third data set [10] contains data as they could be found in meta-analyses of observational studies. The data are from 19 studies with a dichotomous outcome, like in the first data set. However, this time there are no per-group data available for each study but only the comparative association measures (odds ratios) and their standard errors.

**Table 1:** Overview of the data sets used in the criterion validation

**No**	**Author(s)**	**Date**	**Studies**	**Input type**
1	Teo et al.[8]	1991	16	Group size, events
2	Wahlbeck et al.[9]	2000	11	Group size, mean, standard deviation
3	Pagliaro et al.[10]	1992	19	Association measure, standard error

For each data set, we compared the combined association measures, tests for heterogeneity, and tests for small study effects (publication bias) derived from each of the studied meta-analysis programs. We focused on the most common association measures such as the risk difference, risk ratio, odds ratio, mean difference, Hedges' g, and Cohen's d, including their 95% confidence intervals. We used the metan (version 1.81) [14], metabias (version 1.4.2) [15], and metatrim (version 1.5.1) [16] programs of the general statistics software STATA [17] as 'reference' in the software comparisons.

### Assessment of usability

Finally, we performed a usability assessment amongst 30 researchers from various institutes and countries: Kitasato University (Japan), Tokai University (Japan), Utrecht University (The Netherlands), University of Amsterdam (The Netherlands), the Dutch Cochrane Center (The Netherlands), the University of Leuven (Belgium), and the Centre for Statistics in Medicine (UK). There were no specific inclusion criteria and the sample consisted of individuals from various departments and with various levels of experience with meta-analysis.

During the assessment sessions, participants were asked to install (evaluation versions of) each of the studied meta-analysis programs and to analyze one small data set of a meta-analysis with a dichotomous outcome (a shortened version of the previously described meta-analysis by Teo et al. [8]). As they completed this task, they scored the usability of each program in an electronic scoring list. This list [see Additional file 1] was developed via a consensus session with (meta-analysis) experts from the disciplines of epidemiology, biostatistics, and medical informatics, who were asked which elements they considered important in meta-analysis software and what items they would use to judge its usability. The order in which each program was installed and assessed was determined by a computer generated randomization list and different for each participant.

## Results

### Software search and selection

We found 10 meta-analysis packages that were available for download or purchase via the internet (Table 2). Many were no longer updated or had remained in their DOS stage and were excluded from our study. We included six programs in our comparison: Comprehensive Meta-analysis (CMA) Version 2 [18], MetAnalysis[19], MetaWin 2.1 [20], MIX 1.5 [21], RevMan 4.2.8 [22], and WEasyMA 2.5 [23] (in alphabetical order). Using less stringent inclusion/exclusion criteria did not change this software selection. Using more stringent criteria would exclude WEasyMA as various signs indicate that it may no longer be developed and supported. Initially, our search did not pick up the still relatively unknown program called MetAnalysis. This software comes with a book and cannot be purchased separately. Neither the software nor the book is supported by a website, which is why we did not find it at first. At the time of inclusion, we could no longer assess it in the usability part, but have included it post-hoc in the assessment of comparability and features.

**Table 2:** Retrieved meta-analysis software

**Software name**	**OS requirements**	**Meta-analysis interface**	**Availability**	**Selected for review**
Comprehensive Meta-Analysis	WINDOWS	Graphical	Commercial	
EasyMA	DOS	DOS menu	Free	
EpiMeta	DOS	DOS menu	Free	
Hepima	DOS	DOS menu	Free	
Meta-Analysis 5.3	DOS	DOS menu	Free	
Meta-Analyst	DOS	DOS menu	Free	
MetAnalysis	WINDOWS	Graphical	Commercial	
Meta-Stat	DOS	DOS menu	Free	
MetaWin	WINDOWS	Graphical	Commercial	
MIX	WINDOWS	Graphical	Free	
RevMan	WINDOWS	Graphical	Free	
WEasyMA	WINDOWS	Graphical	Commercial	

### Assessment of numerical and graphical features

Below is a short summary of the numerical and graphical features in each of the reviewed programs; details are available in Tables 3 and 4.

**Table 3:** Meta-analysis software – basic feature comparison

	**CMA**	**MetAnalysis**	**MetaWin**	**MIX**	**RevMan**	**WEasyMA**
**General**						
URL	*meta-analysis.com*	*-*	*metawinsoft.com*	*mix-for-meta-analysis.info*	*cc-ims.net/RevMan*	*weasyma.com*
Corporate single user price	~$1295.00	~$75.00	~$150.00	Free	$650	~$490.00
Student single user price	~$395.00	~$75.00	~$75.00	Free	Free	~$280.00
Download/program size	30 Mb	5 Mb	9 Mb	20 Mb/50 Mb	9 Mb	3 Mb
Compatibility	Windows	Windows	Windows	Windows	Windows	Windows
Last update	2006	2005	2002	2006	2005	2002
License	Single user	Single user	Single user	Open	Open	Single user
						
**Input options**						
Manual input						
Copy & paste					()	
Text file import						
File import (Excel, other software)						
Descriptive dichotomous, e.g. n(total), n(y = 1)						
Descriptive continuous, e.g. n, m, sd						
Comparative, e.g. theta, se/var						
Multi-format (mixed in one data set)						
Single data input/selection			()			
Maximum number of studies	Unlimited	Unlimited	Unlimited	100	Unlimited	Unlimited
						
**Information sources**						
Within-program HTML help				()		()
Printable manual						
Description of methods/calculations		()		()		
Additional information sources (PDFs/tutorials)						
Up-to-date website						
						
**Export options**						
Copy output to clipboard						
Export to office application(s)						
Report creation						
Setting copy file type (e.g. bmp, jpg or wmf)						

The ' ' indicates the presence and no mark indicates the absence of a feature. The '()' means that the feature is limited or partially in development, and the '' means it was not working correctly at the time of our assessments.

**Table 4:** Meta-analysis software – analytical feature comparison

	**CMA**	**WEasyMA**	**MetaWin**	**MetAnalysis**	**RevMan**	**MIX**
**Computational setting options**						
Number of decimals						
Alpha level/confidence intervals						
Constant continuity correction						
Treatment arm continuity correction						
Variance for mean differences						
Variances for standardized mean differences						
Bootstrap confidence intervals						
						
**Numerical output**						
Individual study data	AM,CI,P,W,other	AM,CI,W,other	AM,other	AM,CI,other	AM,CI,P,W,other	AM,CI,P,W,other
Association measures – risk	RD,RR,OR	RD,RR,OR	RD,RR,OR	RD,OR	RD,RR,OR	RD,RR,OR
Association measures – means & standardized measures	MD,HG,CD,other		HG,other		MD,HG	MD,HG,CD
Association measures – other	CC,Z					CC
Fixed effect models/weighting	IV,MH,PETO	IV,MH,PETO,other	IV,MH,PETO	IV,MH,PETO	IV,MH,PETO	IV,MH,PETO
Random effects models/weighting	DL	DL	DL	DL	DL	DL
Cumulative analyses	Several variables	Several variables	Several variables	() Only graph		Several variables
Heterogeneity	Q,I^2^,t^2^	Q	Q	Q,I^2^	Q,I^2^	Q,I^2^,t^2^,other
Small study effect/publication bias	FSN,RC,EGG,TF	EGG	FSN,RC	FSN,EGG		FSN,RC,EGG,MAC,TF
Meta-regression	Single moderator		Single moderator			
						
**Graphical output**						
Forest plot						
- Points proportional to weights						
- Annotations in rows possible						
- Cumulative possible						
Funnel plot (1/se, se, var, N, P)	1/se,se	1/se,se,N	var,N	N	1/se	1/se,se,N,P
Galbraith plot				(radial)		
Exclusion sensitivity plot						
Trim and fill plot						
L'Abbe plot						
Other plots			HIST,NQ			BOX,HIST,NQ,other
Graph formatting						

The ' ' indicates the presence and no mark indicates the absence of a feature. The '()' means that the feature is limited or partially in development, and the ' ' means it was not working correctly at the time of our assessments. Abbreviations: AM = association measure, CI = confidence interval, P = P value, W = weight, RD = risk difference, RR = risk ratio, OR = odds ratio, md = mean difference, hg = Hedges' g, cd = Cohen's d, CC = correlation coefficient, Z = Fisher's Z, IV = inverse variance weighting, MH = Mantel-Haenszel weighting, PETO = Peto's weighting, DL = Dersimonian & Laird weighting, Q = Cochran's or Breslow & Day's Q, I^2^=Higgins's inconsistency statistic, t^2^=between study variance indicator, FSN = fail-safe number test, RC = rand correlation test, Egg = Egger's regression test, Mac = Macaskill's regression test, TF = trim and fill method, se = standard error, var = variance, N = sample size, TFP = trim and fill plot, HIST = histogram, NQ = normal quantile plot, BOX = box-and-whiskers plot.

Comprehensive Meta-Analysis (commercial software) has the highest profile in the Internet search engines of all included programs. It distinguishes itself from other programs by the option to enter effect sizes of different formats and the comprehensiveness of the numerical options and output. Data can be entered manually or via copy-and-paste in the CMA spreadsheet; direct import of text or other data files is not possible. The program features all major graphical presentations. The tutorial and manual are to-the-point and extensive. The program is actively maintained and the website is modern and regularly updated.

MetAnalysis 1.0 (commercial software) is not sold separately, but comes as a bonus feature of a book [19]. It is limited to studies with descriptive data on dichotomous outcomes. Data cannot be pasted or imported and must be entered manually, cell by cell. Once the data are entered and the calculations performed, numerical data can be produced in a print preview screen and graphs in separate windows. A nice feature is the radial part of the Galbraith plot, which is lacking in most other software. The software also has the facilities to enter loss to follow-up/drop-out information and use the studies in the meta-analyses with per-protocol or intention-to-treat analysis. The software does not contain help files and does not have a website, but users can consult the book instead.

MetaWin 2.1 (commercial software) is accompanied by a comprehensive manual in the form of a book and, in this respect, resembles the MetAnalysis package described above. Distinctive features are the effect size calculator, some graphs that are relatively uncommon in meta-analysis (the normal quantile plot and a weighted histogram), and the option to use bootstrap confidence intervals. The interface resembles a spreadsheet program and various data files can be imported. For some changes in the analysis, data range selections have to be repeated, which is somewhat more time-consuming compared to methods used by other programs. In contrary to most other software, all calculations are based on t-distributions and boot-strap methods are also available. The help files and the book are extensive and detailed.

MIX 1.5 (free software) is the most recently developed program. Its most prominent features are the comprehensive graphical output, detailed numerical options, and educational features like built-in data sets corresponding to those in a number of books, and extensive tutor functions. MIX is the only program that will not function by itself and it requires Microsoft Excel 2000 or later to run. Another limitation is the maximum number of data sets, which is currently 100. Data sets can be created by manual input as well as by importing text delimited data files or Excel workbooks. The numerical and graphical options are diverse and comprehensive.

RevMan 4.2.8 (free for private and academic use) was developed by and for the Cochrane Collaboration. It stands out due to its extensive features for collaborative management of systematic reviews. The analytical functions of the program cannot be accessed without first creating a review structure and because import and copy-and-paste functionality are also limited, getting started requires more preparation than with most other software. Once data are in the analysis module, analysis is straightforward. Output is detailed, though without tests for publication bias and no other graphs than the forest and funnel plot. The help resources in RevMan are extremely thorough. A new version is to be released in the near future.

WEasyMA 2.5 (commercial software) stands out by the speed with which results become available after data set creation. Data cannot be imported or pasted and need to be entered manually, cell by cell. Another limitation of this program is that it can only handle data from clinical trials with dichotomous outcomes, e.g. two-by-two table data. Although limited to these types of data, the program produces a wide variety of numerical and graphical output. The original author has indicated that the software is currently unsupported by a development team and may soon no longer be available.

### Validity and comparability of meta-analysis results

Our internet and database search did not yield any publications on the validity or validation of any of the programs, except for MIX [24,25]. Authors of all programs were contacted to determine whether (yet unpublished) evidence of validation procedures was available. Authors of RevMan indicated that validation data were made public via notes and abstracts at Cochrane Collaboration meetings and conferences. The authors of CMA, MetAnalysis, and MetaWin stated that all procedures had been checked extensively with external programs, spreadsheets, and occasionally by hand, though had not been made public. For CMA, Excel sheets with such data are available upon request. We received no information on validation procedures from the authors of WEasyMA.

We found no discrepancies in meta-analysis results between STATA, MIX and RevMan. In CMA, we found a small inconsistency in results of publication bias tests, but this was corrected via an update while we were writing this article.

MetaWin's results were different from STATA's results (and thus also from results in CMA, MIX, and RevMan) because MetaWin mostly uses a t-distribution where the aforementioned programs use a z-distribution (although a recent version of MIX also allowed us to use a t-distribution). We did find what seemed to be a terminological inconsistency, as the Mantel-Haenszel labeled method used in MetaWin for odds ratio analyses gave results that were identical to those from Peto's method in the other programs (albeit with confidence limits based on a t-distribution).

Since MetAnalysis and WEasyMA can only analyze data from two-by-two tables, the comparability assessments were limited to one data set [8]. Analyses in MetAnalysis were very similar though not always identical to those from STATA. We found that if we entered experimental group data first (as is the case in all other software), an incorrect event coding is applied that causes the software to calculate risk differences and odds ratios of survival even if mortality is entered as event. For risk differences this only changes the sign, but for odds and odds ratios it gives the reciprocal of the intended results [26]. Although the book mentions that control data are to be entered in the first data column, the software has currently no built-in guard against this and we therefore urge users to be careful.

In WEasyMA, we found results that could not be reproduced if a data set with zero events in one study arm was used. Even when using the same continuity correction as reported in the 'Calculation options' dialog in WEasyMA, the results remained different in STATA. The WEasyMA authors did not respond to our inquiry into reasons for the discrepancies.

### Assessment of usability

Of the 30 participating researchers, 26 provided quantitative data that were suitable for analysis (Table 5). Trouble with the electronic user form or installation of software made the data from 4 researchers incomplete and they were excluded from the quantitative part. MIX scored highest on the overall usability (8.6), followed by CMA (6.9), MetaWin (6.2), RevMan (6.1), and WEasyMA (4.2).

**Table 5:** Meta-analysis software – usability ratings (best scoring software from left to right)

**Items and subgroups**	**MIX**	**CMA**	**MetaWin**	**RevMan**	**WEasyMA**
**All researchers (26)**					
Overall rating (min-max)	8.6 (6.7 to 10)	6.9 (3.7 to 9.7)	6.2 (4.3 to 8.7)	6.1 (4.3 to 8.3)	4.2 (1 to 7.3)
Getting started	8.6	7.4	6.8	7.6	4.5
Data preparation	8.3	6.3	6.3	4.5	2.6
Usability in analysis	8.8	7.1	5.6	6.3	5.9
					
**Experienced (7)**					
Overall rating (min-max)	8.1 (7.0 to 9.7)	6.8 (6.0 to 7.3)	5.9 (4.3 to 7.7)	5.4 (4.3 to 6.3)	3.3 (1 to 5.7)
Getting started	8.0	7.6	6.2	7.5	2.8
Data preparation	8.3	6.3	6.3	3.0	2.0
Usability in analysis	8.0	6.6	5.4	6.3	5.3
					
**Inexperienced (19)**					
Overall rating (min-max)	8.7 (6.7 to 10)	7 (3.7 to 9.7)	6.3 (4.3 to 8.7)	6.3 (4.7 to 8.3)	4.6 (1.3 to 7.3)
Getting started	8.8	7.3	6.9	7.7	5.0
Data preparation	8.3	6.3	6.3	5.0	2.8
Usability in analysis	9.1	7.3	5.6	6.3	6.1

All scores are summary scores, based on the scores of items in the 'Installation', 'Data preparation', and 'Usability in analysis' categories. Each item was scored from bad to excellent on a scale from 0 to 10.

RevMan was most familiar to the participating researchers. MIX had not been used by any of the participants but the name was familiar to some as they were affiliated to the same institutions as the makers of the MIX software. Stratifying the results in analogous subgroups did not reveal any specific trends in the ratings. Experienced users appeared to be more critical than less experienced users, but relative scores were identical. Installation of WEasyMA and CMA was troublesome for some researchers. Qualitative statements mostly concerned problems with the installation (WEasyMA, CMA), error messages in French (WEasyMA), and difficulties with data set creation (WEasyMA, RevMan). Favorable comments included praise for the user interfaces (MIX, RevMan, CMA), help system (RevMan), speed of analysis (WEasyMA), and within-program tutoring (MIX, CMA).

## Discussion

Meta-analysis is an indispensable tool in current-day synthesis of research data from multiple studies, and systematic reviews with meta-analyses occupy the top position in the hierarchy of evidence. Software for meta-analysis has evolved over the years and available reviews are relatively outdated. We therefore considered it timely to provide a systematic overview of the features, criterion validity, and usability of the currently available software that is dedicated to meta-analysis of causal (therapeutic and etiologic) studies. It has some overlaps with existing reviews [3-7], but includes other more recent programs, contains more detailed information on the merits and demerits of the available programs, and follows a more systematic approach.

We studied four commercial programs (CMA, WEasyMA, MetaWin, and MetAnalysis) and two free programs (RevMan and MIX). The features of the commercial programs were not necessarily more extensive than those of the free ones. In particular MIX stood out in terms of numerical options and graphical output. CMA was generally most versatile, in particular in options for analysis of various types of data. With regard to the comparability of results, MIX, RevMan, and CMA produced numerical results that were identical to results from STATA's metan, metabias, and metatrim. MetaWin's results are different and slightly more conservative, since the confidence intervals are based on a t-distribution or bootstraps. WEasyMA produces results that can be disparate from the other programs, especially in data sets with studies with zero events in one or both of the comparison groups. Although most differences were small in the data sets we used, we have reservations on how this will reflect on data sets with more extreme data. The MetAnalysis program should also be used with care as data have to be entered manually and in the correct columns. Exchanging the columns is currently not prevented by warning or error messages and can lead to invalid results.

The usability study shows that preparing data for analysis is the hardest part in each program. MIX and CMA are identified as the most user-friendly programs. WEasyMA scored least favorable. Stratifying user evaluations based on experience with meta-analysis and previous experience or knowledge of the software did not reveal any trends in the ratings.

Our comparison has been limited to software dedicated to meta-analysis only and does not include general statistics packages. The primary reason to leave them out was because they are structurally very different, making direct comparisons inappropriate. Central to this issue is software syntax: most general packages require thorough knowledge of their syntax in order to produce and alter graphs that are common in meta-analysis; the dedicated packages, however, produce such graphs with a few or sometimes even a single click. In addition, the syntax knowledge required to do more advanced meta-analyses with the general packages means that in a usability survey all participants would have to be expert statisticians, capable of writing and adapting syntax for meta-analysis in all major general software packages. This is not only not feasible in the current setting, it would also make the participating individuals no longer representative of the (sometimes relatively inexperienced) users of the software in the scientific and academic community. Although a different approach would be necessary, we believe the user community of meta-analysis software would benefit from an additional review of meta-analysis options in general statistics software.

Due to the lack of a 'gold' standard, we resorted to between-program comparisons and a criterion validation with STATA's user-written commands *metan*, *metabias *and *metatrim *as reference. Our choice for STATA was based on its versatility and use in two major books on meta-analysis [11,12]. We realize that STATA itself is also user-written and potentially subject to similar validity issues than the other programs. The fact that CMA, MIX, and RevMan produced results that were identical to results from STATA, at least with the three data sets we selected, justifies to some extent our use of STATA as a reference standard.

The results of our usability survey should be regarded as exploratory and serve as a rough indication. First, the number of participants was relatively small. Second, it is not unlikely that there may be some bias in favor of RevMan and MIX because some users were already familiar with these programs. Subgroup analyses, however, did not reveal such trends. MetAnalysis could unfortunately not be included as it was included after the start of the usability assessment. A further point regarding MIX is that it was created following a development focus list [25] that was created in a similar fashion to our usability scoring list. Assessment of both lists reveals that a number of items are very similar. Although this may indicate that the lists are indeed reflecting the demands of statistical software users, it also means that the MIX program was likely to do well in our assessment. We believe, however, that any program that is systematically developed to satisfy its users' demands should perhaps deservedly score high.

Another point to which we would like to draw attention is the lack of accessible public information about the manner in which meta-analysis programs have been validated. Only the website of the MIX program includes specific references to this and MIX is the only program with a peer-reviewed and published validation report [25]. Without such reports, authors, reviewers, editors, and consumers of evidence have no reference for judgments about the suitability of the software for scientific purposes. This is of course equally applicable to the user-written meta-analysis macros for general statistics software. We argue for more rigor and transparency in this area.

Finally, we are fully aware that the world of information technology changes constantly and by the time this manuscript is published, it is possible that some updates have become available or that new products have been launched. We apologize beforehand for our lack of timing. Like a traditional review, we intend to update this investigation in due time.

## Conclusion

In conclusion, the most suitable meta-analysis software for a user depends on his or her demands; no single program may be best for everybody. The information provided in this article, in particular the data in Tables 3 and 4, should give users the opportunity to make a substantiated decision.

## Competing interests

None of the authors have financial conflicts of interest, although the first author is also primary developer of one of the free programs (MIX) studied in this review. The other authors have been co-authors in an introductory article about MIX. To reduce personal biases, all tasks were handled by multiple investigators and the subjective usability assessments were assigned (by study design) to individuals other than the authors.

## Authors' contributions

LB and KGM developed the study concept and designed the study. LB and LMY handled the primary data acquisition and drafted the manuscript. All authors double checked the data tables and analyses, and approved the final version of the manuscript.

## Pre-publication history

The pre-publication history for this paper can be accessed here:



## Supplementary Material

Additional file 1 Software usability scoring list. The scoring list that was used to evaluate the usability of the meta-analysis software. Click here for file
