# Supplementary material for: A systematic comparison of software dedicated to meta-analysis of causal studies
Source: BMC Med Res Methodol. 2007 Sep 10;7:40. doi: 10.1186/1471-2288-7-40 (PMC2048970; doi:10.1186/1471-2288-7-40)
Supplement: Additional file 1 — Software usability scoring list. The scoring list that was used to evaluate the usability of the meta-analysis software. [file 1471-2288-7-40-S1.doc]

***Software usability scoring list***

| *Score each item on a scale from 1 to 10 and provide overall and per-category summary scores* |  | |
| --- | --- | --- |
| **GETTING STARTED** | **Score (1-10):** | |
| Installation time *(Fast or slow?)* | |  |
| Desktop icons installed (installable) *(Possible via installer program?)* | |  |
| Software introduced *(Introductory explanation of usage?)* | |  |
| Requirement of manual actions *(All files and icons automatically in the right place?)* | |  |
| Uninstaller available (Start menu) *(Uninstall program available in Start > Programs?)* | |  |
|  | | |
| **DATA PREPARATION** | **Score (1-10):** | |
| Program start *(Speed? Errors? Help? Intuitiveness of start-screen?)* | |  |
| Program data import / reading *(Import CSV/text files? Paste data set?)* | |  |
| Preparation of data for analysis *(Easy? Speed?)* | |  |
| Data creation speed *(Could you create a data set quickly?)* | |  |
| Guidance *(Availability of help for data set creation?)* | |  |
|  | | |
| **USABILITY IN ANALYSIS** | **Score (1-10):** | |
| General help *(Availability of help functions? Useful?)* | |  |
| Intuitiveness of interface *(General interface design? Menus & toolbars where you want them?)* | |  |
| Ease of numerical output creation | |  |
| Ease of graphical output creation | |  |
| Adaptability of graphs *(Possible via menu, double-click, or right-click? Options?)* | |  |
| Ease of analysis changes | |  |
| Ease of output copy / export for reports *(Right-click copy? File types? Export or report functions?)* | |  |
|  | | |
|  | | |
|  | **Score (1-10):** | |
|  | | |
